# Adolescents’ Sexual Wellbeing in Southwestern Uganda: A Cross-Sectional Assessment of Body Image, Self-Esteem and Gender Equitable Norms

**DOI:** 10.3390/ijerph15020372

**Published:** 2018-02-22

**Authors:** Elizabeth Kemigisha, Viola N. Nyakato, Katharine Bruce, Gad Ndaruhutse Ruzaaza, Wendo Mlahagwa, Anna B. Ninsiima, Gily Coene, Els Leye, Kristien Michielsen

**Affiliations:** 1 Faculty of Interdisciplinary Studies, Mbarara University of Science and Technology, Mbarara 1410, Uganda; vnyakato@must.ac.ug (V.N.N.); kbruce2@tulane.edu (K.B.); gruzaaza@must.ac.ug (G.N.R.); wolema@must.ac.ug (W.M.); 2 International Centre for Reproductive Health, Faculty of Medicine and Health Sciences, Ghent University, 9000 Ghent, Belgium; els.leye@ugent.be; 3 RHEA, Centre of Expertise on Gender, Diversity and Intersectionality, Vrije Universiteit Brussel, 1050 Brussels, Belgium; annekiiza2001@yahoo.com (A.B.N.); gily.coene@vub.ac.be (G.C.)

**Keywords:** self-esteem, gender norms, body image, young adolescents, Uganda, sexuality, sexual health

## Abstract

Measures of sexual wellbeing and positive aspects of sexuality in the World Health Organization definition for sexual health are rarely studied and remain poorly understood, especially among adolescents in Sub-Saharan Africa. The objective of this study was to assess sexual wellbeing in its broad sense—i.e., body image, self-esteem, and gender equitable norms—and associated factors in young adolescents in Uganda. A cross-sectional survey of adolescents ages 10–14 years in schools was carried out between June and July 2016. Among 1096 adolescents analyzed, the median age was 12 (Inter-Quartile Range (IQR): 11, 13) and 58% were female. Self-esteem and body image scores were high with median 24 (IQR: 22, 26, possible range: 7–28) and median 22 (IQR: 19, 24, possible range: 5–25) respectively. Gender equitable norms mean score was 28.1 (SD 5.2: possible range 11–44). We noted high scores for self-esteem and body image but moderate scores on gender equitable norms. Girls had higher scores compared to boys for all outcomes. A higher age and being sexually active were associated with lower scores on gender equitable norms. Gender equitable norms scores decreased with increasing age of adolescents. Comprehensive and timely sexuality education programs focusing on gender differences and norms are recommended.

## 1. Introduction

The World Health Organization (WHO) defines sexual health as “a state of physical, mental and social wellbeing in relation to sexuality. It requires a positive and respectful approach to sexuality and sexual relationships, as well as the possibility of having pleasurable and safe sexual experiences, free of coercion, discrimination and violence” [1]. Sexual wellbeing aspects in the current WHO definition for sexual health are rarely explored. Most scholars, country demographic surveys and international reports focus on the negative aspects of sexual health and risky sexual behavior. Understanding positive sexual health attributes is important as these empower young people to make informed choices for better sexual and reproductive health (SRH) outcomes [2]. This is particularly important among young adolescents (10–14 years) who are commonly neglected in research. Young adolescents are at a formative stage of development, but often ill-equipped with relevant knowledge and decision-making skills. This period is important to laying a foundation for positive SRH behaviors and outcomes [3,4].

As a developing country in which adolescents comprise 1/3 of the total population, Uganda is facing serious challenges related to SRH of young people [5]. These challenges include: low comprehensive Human Immunodeficiency Virus and Acquired Immune Deficiency Syndrome (HIV/AIDS) prevention knowledge at 40% with heightened risks for Sexually Transmitted Infection (STI) acquisition, high teenage pregnancy at 25% which contributes 24% of maternal mortality, and low contraceptive uptake [6]. However, there is a paucity of studies in Sub-Saharan Africa that describe positive attributes of adolescents’ sexual health, which are pertinent to address prevailing SRH challenges.

Recently, there has been growing attention among scholars on the assessment of adolescent sexual wellbeing [2,7]. Recent work by Russel (2005) and Blum et al. (2014) emphasizes the need for a socio-ecological framework to assess adolescent sexuality at the individual, institutional and societal or cultural levels [3,8]. Furthermore, Harden (2014) explores the possibility of evaluating adolescent sexual wellbeing based on factors such as adolescent self-efficacy, sexual self-esteem, sexual pleasure and satisfaction, and freedom from pain and negative effects related to sexuality [9]. These aspects have been further emphasized by the European expert group on sexuality who also prioritize evaluation of aspects related to positive attitudes towards gender equality and adolescent empowerment in making informed choices [2]. Few studies have evaluated gender equitable norms, self-esteem and body image or satisfaction as they relate to positive outcomes of sexual and reproductive health [10,11,12,13]. There is no consensus on how aspects of sexual wellbeing should be assessed and reported, especially among the adolescent population, and the research on this topic is limited [2,7].

This study assessed sexual wellbeing of young adolescents in primary schools in Southwestern (SW) Uganda. We looked at several aspects related to wellbeing, namely self-esteem, gender equitable norms and body image scores, as well as their associations with socio-demographic characteristics, school and family environment, and sexual activity. We focused on individual factors such as gender, age, educational level and socio economic status as well as interpersonal and environmental factors such as school absenteeism, bullying, number of parents alive, exposure to media and sexual onset, as these have been shown to influence adolescent SRH [14]. We hypothesized that there would be a difference in scores for sexual wellbeing among younger adolescents (10–12 years compared to 13–14 years) and higher scores among boys compared to girls [15].

## 2. Materials and Methods

### 2.1. Study Design and Sample Population

This study utilized a cross-sectional design. The data was collected as part of a cluster randomized trial analyzing the effectiveness of a comprehensive sexuality education intervention among young adolescents ages 10–14 years in primary schools in SW Uganda. The sample size was 1104 adolescents in primary 5 and 6 drawn from 33 schools in urban and rural settings in Mbarara district.

### 2.2. Main Outcomes

The main outcomes for the study were self-esteem, body image and gender equitable norm scores, which were used independently as proxy indicators for positive aspects of sexual health. Self-esteem scores were estimated using 7 of 10 items of the Rosenberg (1965) self-esteem scale [16]. These included positively scored items such as; “*I feel that I am a person of importance*”; and negatively scored items such as “*I feel useless at times.*” Item 4 “*I can do things as well as most people*,” Item 8 “*I wish I could have more respect for myself*” and Item 10, “*I take a positive attitude toward myself*” were excluded. Items 8 and 10 were excluded because they were complex and not suitable for the age group, whereas Item 4 was excluded at analysis due to poor correlation with other items in the scale. The Cronbach alpha reliability test for the items was low but acceptable at 0.60 [17]. Body image scores were estimated using 5 of the 6 items of the Body Image States Scale (BISS-6) [18]. These included items such as “*Right now, I feel extremely satisfied with my looks.*” Item 5 “*Right now, I feel much worse about my looks than I usually feel*” was excluded at analysis due to poor correlation with the rest of the items on the scale, and the scale was modified from a 9 points to a 5 points Likert scale for ease of use for this age group. The Cronbach alpha reliability test for the items was 0.67. Gender equitable norms scores were estimated using 11 items. Six of these items were adapted from the Attitudes towards Women Scale for Adolescents (AWSA) [19] and 5 items were developed to suit the respondents’ age and the Ugandan context. Items from the AWSA scale included, “*On average boys are cleverer than girls*” and “*Girls should be free to go out unaccompanied to meet with friends*”*.* The other items developed to relate to the Uganda context included, “*If there is a sick person at home, only a girl should stay home to care for the sick one as the boy goes on with school*” or “*My mother more than my father should be blamed for my mistakes.*” The items were scored on a 4 point Likert scale. The Cronbach alpha reliability test for the items was 0.68. Individuals with missing data on any of the items on a scale were eliminated from that analysis.

### 2.3. Correlates

Information on social and demographic variables such as age and gender were collected. Pubertal or Tanner stage was estimated by self-assessment, in which respondents were shown a pictorial representation of the Tanner stages and told to select the image that best represented their own stage of development. This provided estimates of pubertal stages without requiring more invasive inspections that often make young adolescents feel uncomfortable. Studies such as Rasmussen et al. 2015 have shown that although self-assessment is not a reliable measure of exact pubertal timing, it is a sufficient estimate of relative pubertal stage for large epidemiologic studies [20].A socio-economic score was developed based on household water source, distance from water source, household possessions and pupil possessions including shoes and pairs of school uniforms. We developed this score using a subset of items from the standard Demographic and Health Survey score so that the data could be more readily collected from young adolescents, as opposed to using other standard measurements which are administered to adults. Students self-reported on truancy and sexual activity. Truancy was defined as absence from school for any reason other than that the student was sick. Sexual activity was defined as a student reporting having had penetrative vaginal sex. Exposure to visual or audiovisual media of sexual content (pornographic content) was also assessed.

### 2.4. Statistical Analysis

Data analysis was done using Stata^®^ (College Station, TX, USA). Continuous variables were summarized using means (SD) and medians (IQR) whereas categorical variables were summarized using proportions (percentages). Mean (SD) was used to summarize gender norms scores because they were normally distributed, while medians (IQR) were used to summarize body image and self-esteem because they were non-normally distributed. Among categorical variables, a bivariate analysis was conducted using odds ratios. Among continuous variables, a *t*-test was conducted for variables that were normally distributed, and a Mann–Whitney test was conducted for variables that were non-normally distributed. Multivariate logistic regression analysis with stepwise variable selection was performed on variables with a *p*-value less than 0.2 at bivariate analysis to assess the association between self-esteem scores ≤ median vs. > median, body image score ≤ median vs. > median, and gender equitable norms mean scores with socio-demographic, school and family factors and sexual behavior, respectively. A test for collinearity was done prior to multivariate analysis and for all the models the Variance Inflation Factors were less than 1.5. Odds ratios and 95% confidence intervals were calculated. A *p*-value of less than 0.05 was considered to reflect a significant association.

### 2.5. Ethics

The study was approved by the Mbarara University Research Ethics Committee (reference MUIRC 1/7) and the Uganda National Council for Science and Technology (reference SS 4045). We also obtained ethical approval from Ghent University as a partner institution. Written parental consent, school head teacher consent and pupils’ assent were obtained prior to data collection.

## 3. Results

### 3.1. Description of the Study Population

Between June and July 2016, a total of 1104 primary school pupils ages 10–14 were interviewed. Of these, 8 were excluded because their age could not be verified, and 1096 were included in this analysis. Among the 1096 adolescents, 460 (42%) were male, the median age was 12 years (IQR: 11, 13), 635 (57.9%) were between ages of 10–12 years, and 896 (81.8%) were from schools in rural areas. A total of 79 (17.3%) pupils reported bullying and 249 (22.7%) reported truancy, as defined by absence from school for any reason other than illness. Only 83 (7.6%) reported being sexually active, 67 (80.7%) of which were boys (Table 1).

### 3.2. Outcomes

#### 3.2.1. Self-Esteem

The median self-reported self-esteem score was 24 (IQR: 22, 26, range: 11–28) with a possible score range of 7–28. Girls had higher scores compared to boys, with 282 (46.4%) girls and only 153 (34.6%) boys scoring above the median. A Mann–Whitney test found that the difference in median self-esteem scores between each gender was statistically significant (*p* < 0.0001).

#### 3.2.2. Body Image

The median self-reported body image score among respondents was 22 (IQR: 19, 24, range: 8–25) with a possible score range of 5–25. Girls reported higher scores than boys, with 302 (47.8%) girls and only 174 (38.2%) boys scoring above median. A Mann–Whitney test found that the difference between median scores for boys and girls was statistically significant (*p* = 0.002).

#### 3.2.3. Gender Equitable Norms

The mean score for gender equitable norms was 28.1 (SD 5.2; range: 13–44), with a possible score range of 11–44. Girls had higher scores on gender equitable norms with a mean of 29.1 (SD 4.9) compared to 26.7 (SD 5.3) in boys. A *t*-test found that the difference in mean scores for boys and girls was significant (*p* < 0.00001).

### 3.3. Bivariate Analysis of Factors Associated with Main Outcomes

Results of bivariate analysis are presented in Table 2. Female gender was associated with higher scores for gender equitable norms, self-esteem and body image. Other characteristics associated with higher self-esteem scores included being of higher education level and religious denomination, that is, being of Catholic faith compared to Moslem or Anglican. Alternatively, pupils from urban schools and those who were truant (absent from school without clear reason) overall had lower self-esteem scores. Higher scores on gender equitable norms were reported among those with a higher socio-economic status, a higher tanner stage and a younger age. Pupils with lower gender equitable norms scores were more likely to have ever had sex. Furthermore, pupils in a higher education level and those from urban schools were more likely to have higher scores on body image.

### 3.4. Multivariate Analysis of Factors Associated with Main Outcomes

Results of multivariate analysis are presented in Table 3. On multivariate analysis with key outcomes, female gender was associated with increased scores for self-esteem, body image and gender equitable norms. Furthermore, being in primary 6 as opposed to primary 5 was associated with higher scores for body image and self-esteem, but had no association with gender equitable norms. Older age, higher Tanner stage, lower socio-economic status, and having both parents alive compared to only one and none were associated with lower scores on gender equitable norms. Additionally, early sexual activity was associated with lower gender equitable norms scores. However, no associations were established between scores for body image or self-esteem and sexual activity. Pupils who reported school absence without a reason were more likely to have lower self-esteem scores. Students from urban schools were more likely to have lower self-esteem scores but higher body image scores.

## 4. Discussion

Few validated measures exist for assessing positive aspects of sexual health among young adolescents, especially in the cultural context of Sub-Saharan Africa. This is one of the first studies in Uganda to measure sexual wellbeing in this subpopulation, using the proxy indicators of gender equitable norms, self-esteem, body image and relevant covariates. Overall, this research found high scores for body image and self-esteem but moderate scores for gender equitable norms among young adolescents in SW Uganda. The study also provides insight into individual and interpersonal factors at the family and school levels that are associated with body image, self-esteem and gender equitable norms. Findings illustrate diminishing gender equitable norms with increasing age and an association between low gender equitable norm scores and early sexual onset. However, we did not establish in this study any associations between scores for body image or self-esteem and sexual activity.

We found moderate scores on gender equitable norms for young adolescents. This indicates that adolescents in Uganda are already socialized at a young age to have inequitable norms, a finding which has been described in another recent study [21]. In addition, our finding that early sexual onset was associated with having lower gender equitable norms scores is of concern, as there is existing evidence that equitable gender norms promote good sexual outcomes [10,22,23]. These findings, together with the association between lower gender equitable norms scores and increasing age, are of paramount importance as they relate to the timing of educational interventions to promote gender equality. Furthermore, the finding that girls had higher gender equitable norms scores than boys has important implications for educational interventions. These gender disparities could be explained by the fact that most mainstream media and health promotion programs in schools that address adolescent SRH vulnerabilities in Uganda focus on protecting and supporting the girl child only while neglecting the needs of boys [24]. The findings of this study, along with other related research in this field, highlight the importance of addressing issues of gender and equality among both boys and girls at a young age.

Additionally, our study found that the presence of both parents compared to one or none was associated with having lower gender equity. Because equitable gender norms are clearly shaped in early childhood, parents play an important role [25,26]. This finding also highlights the fact that gender norms are influenced by multiple levels of the socio-ecological model, and interventions will therefore be most effective if they address multiple levels of the socio-ecological model. Educational interventions should involve parents as well as adolescents in order to produce meaningful and sustainable change.

We found high scores in body image and self-esteem in this age group. The findings that females were more likely than males to have higher body image and self-esteem scores contradicts much of the existing literature on gender, body image and self-esteem, which has often found that male adolescents have higher self-esteem than females [15,27]. This contradiction could be explained by the fact that our study focuses on young adolescents, while other studies on adolescents often include an older age range. It could also be related to the Ugandan context, as most research related to body image and self-esteem is conducted in Western countries [15,27,28]. It is possible that a focus on the girl child, common in many health education programs in Uganda, might contribute to higher scores in body image and self-esteem among girls compared to boys. One study conducted in seven Ugandan schools found that girls received more guidance than boys about their SRH, both in the classroom and in the home [29]. Another study of Ugandan adolescents found that boys were less likely than girls to have discussed SRH issues with their parents [30]. Whether in the classroom or at home, young boys in Uganda are less likely to discuss their SRH with adults, which may contribute to anxiety about their changing bodies and lower self-esteem.

Certain environmental and school factors were found to be associated with high self-esteem and body image scores. Living in an urban vs. a rural area was associated with lower scores on self-esteem but higher scores on body image. Economic disparities within the urban environment could explain the relatively low self-esteem scores in this group. Improving education and narrowing disparities in incomes and social amenities among people living in urban areas could reduce the gap in self-esteem. Regarding body image, it was found that being in the older age group was associated with higher body image scores, and that children from rural schools were significantly older than their counterparts in urban schools. It is therefore possible that the discrepancy in body image scores between urban and rural students may actually be explained by age and not by location. No associations between self-esteem scores or body image scores with sexual onset were established in this study. There is contradicting evidence regarding the association between self-esteem and sexual efficacy or behavior, with a few studies describing no relationship [13,27,31], whereas Ethier et al. 2006 [12] describe a positive association between the two. Studies on body image have been more or less consistent in showing associations with positive sexual outcomes [32]. The lack of an association between self-esteem or body image scores and sexual behavior in our study population could reflect a genuine lack of association. However, it may also be attributable to low variation in scores for body image and self-esteem in this group.

The study had a few key limitations. Due to the cross-sectional nature of this study, it was not possible to determine whether some of the individual or contextual factors preceded the outcome variables. Another limitation was the potential risk of social desirability bias due to self-reporting of behaviors. Whenever possible, interviewers and participants were matched on gender to try to limit this bias. Lastly, we used scales for measurement of gender norms, self-esteem and body image that have mainly been used for older adolescents. We therefore adapted these scales and piloted them. Cronbach alpha scores were slightly lower than what is generally considered acceptable, which might suggest that the results of these scores had lower reliability than would be ideal. However, because Cronbach alpha is a function of the scale length as well as the reliability, it tends to underestimate the reliability of surveys like ours which have relatively few items [33]. It is also possible that the specific items chosen from the Rosenberg self-esteem scale could have influenced the gender discrepancy in self-esteem scores. The Rosenberg scale used in this study may have limitations in its applicability because it is unidimensional. Studies that consider self-esteem from a multidimensional perspective have found that girls score higher on certain components of self-esteem, while boys score higher on others [34,35], meaning that in selecting a subset of items we may have selected items on which girls generally have higher scores. Future research should consider the multidimensionality of self-esteem.

## 5. Conclusions

Young adolescents in primary schools in Uganda have high scores for body image and self-esteem and moderate scores on gender equitable norms. Gender equitable norms scores reduce with increasing age, suggesting that sexuality education interventions need to start while adolescents are still young, before they have developed these harmful norms. Further research is recommended on (1) establishing causal relationships between socio-ecological variables and gender equitable norms, self-esteem or body image, and (2) validation of measurements for attributes of positive sexual health in different contexts among very young adolescents. More studies are needed in the Ugandan setting to explain possible relationships between measures of sexual wellbeing and positive sexual health outcomes in young adolescents.

## Figures and Tables

**Table 1:** Socio-demographic characteristics, family and school factors and sexual behavior of study participants.

	Overall, n (%) n = 1096	Male n (%) n = 460 (42.0)	Female n (%) n = 636 (58.0)	*p*-Value
Age in years				
10–12	635 (57.9)	259 (56.3)	376 (59.1)	
13–14	461 (42.1)	201 (43.7)	260 (40.9)	0.351
Pubertal age (Tanner 1–5)				
Tanner 1 or 2	509 (46.4)	217 (47.2)	292 (45.9)	1
Tanner stage >2	584 (53.6)	243 (52.8)	344 (54.1)	
Education level				
Primary 5	563 (51.4)	240 (52.2)	323 (50.8)	
Primary 6	533 (48.6)	220 (47.8)	313 (49.2)	0.650
Socio-economic status				
Low	315 (29.0)	128 (28.2)	187 (29.6)	
Medium	511 (47.1)	215 (47.4)	296 (46.8)	
High	260 (23.9)	111 (24.4)	149 (23.6)	0.871
Religion				
Moslem	101 (9.2)	48 (10.5)	53 (8.3)	
Anglican/Pentecostal	587 (53.7)	245 (53.5)	342 (53.9)	
Catholic	405 (37.1)	165 (36.0)	240 (37.8)	0.462
Family and school factors				
Location of school				
Rural	896 (81.8)	368 (80)	529 (83.2)	
Urban	199 (18.2)	92 (20)	107 (16.8)	0.178
School absenteeism (truancy)	249 (22.7)	100 (21.7)	149 (23.4)	0.510
Ever experienced bullying at school	79 (17.3)	119 (18.7)	198 (18.1)	0.528
Number of parents alive				
Both alive	905 (82.5)	378 (82.2)	527 (82.8)	
None/Single parent	124 (11.3)	48 (10.4)	76 (12.0)	0.350
Access to media (TV, Social media or Tabloids)				
Television	458 (41.8)	217(47.3)	241 (37.9)	0.002
Tabloid newspapers	222 (20.3)	125 (27.2)	97 (15.3)	<0.0001
Social media	70 (6.4)	27 (5.9)	43 (6.8)	0.558
Ever watched pornographic content	389 (35.5)	191 (41.5)	198 (31.2)	<0.0001
Sexual behavior				
Ever had sex	83 (7.6)	67 (14.6)	16 (2.5)	<0.0001

**Table 2:** Bivariate analysis of self-esteem, body image and gender equitable norms with social and demographic, school and family factors and sexual behaviors.

	Self-Esteem		Body Image		Gender Equitable Norms	
	OR ^†^	95% CI	OR	95% CI	Beta Coefficient	95% CI
Gender						
Male	1					
Female	1.64	(1.28–2.12) ***	1.48	(1.16–1.89) **	2.42	(1.78–3.05) ***
Age						
10–12	1					
13–14	1.06	(0.83–1.37)	1.26	(0.99–1.60)	−0.95	(−1.6–−0.31) **
Pubertal age (Tanner 1–5)						
Tanner 1 and 2	1					
Tanner 3 to 5	1.06	(0.73–1.55)	1.11	(0.77–1.61)	1.52	(0.54–2.51) **
Education level						
Primary 5	1					
Primary 6	1.51	(1.19–1.94) **	1.39	(1.09–1.77) **	0.1	(−0.54–0.74)
Socio-economic status						
Low	1					
Medium	0.99	(0.74–1.32)	1.13	(0.85–1.50)	−0.05	(−0.81–0.70)
High	1.02	(0.73–1.43)	1.23	(0.88–1.71)	1.43	(0.55–2.31) **
Religion						
Moslem	1					
Anglican/Pentecostal	1.49	(0.94–2.35)	1.11	(0.73–1.71)	−0.45	(−1.58–0.68)
Catholic	1.84	(1.15–2.95) *	0.78	(0.50–1.22)	−0.017	(−1.34–0.99)
Location of school						
Rural	1					
Urban	0.62	(0.45–0.87) **	1.46	(1.07–1.99) *	0.6	(−0.21–1.44)
School absenteeism (truancy)	0.66	(0.49–0.89) **	0.87	(0.65–1.15)	−0.2	(−0.97–0.56)
Number of parents alive						
None/Single parent	1					
Both alive	1.11	(0.80–1.54)	1.23	(0.89–1.69)	−0.71	(−1.59–0.17)
Ever watched pornography	0.89	(0.69–1.15)	1.11	(0.86–1.42)	−0.56	(−1.23–0.11)
Ever had sex	0.64	(0.39–1.05)	0.63	(0.39–1.01)	−2.30	(−3.50−1.10) ***

^†^ Odds Ratio * *p* < 0.05; ** *p* < 0.01; *** *p* < 0.001.

**Table 3:** Multivariate analysis of self-esteem scores, body image, gender equitable norm score with socio-demographic, family and school factors and sexual behavior.

	Self-Esteem		Body Image		Gender Equality Score	
	AOR ^‡^	95% CI	AOR	95% CI	Beta Coefficient	95% CI
Gender						
Female	1.65	(1.28–2.14) ***	1.52	(1.19–1.96) **	2.26	(1.62–2.92) ***
Age						
10 to 12 vs. 13 to 14	1.07	(0.82–1.40)	1.39	(1.08–1.79) *	−0.68	(−1.33–−0.03) *
Tanner age						
Tanner 1–2						
Tanner 3–5	0.89	(0.69–1.15)			−0.66	(−1.29–−0.17) *
School location						
Rural vs. Urban	0.61	(0.44–0.86) **	1.61	(1.18–2.22) **	0.58	(−0.22–1.40)
Parent alive						
One/both parents dead						
Both parents alive			1.28	(0.92–1.77)	−0.89	(−1.74–−0.03) *
Socio-economic status						
Low						
Medium					0.05	(−0.68–0.78)
High					1.49	(0.63–2.34) **
Truancy	0.62	(0.46–0.84) **				
Ever had sex					−1.25	(−2.44–−0.05) *

^‡^ Adjusted Odds Ratio * *p* < 0.05; ** *p* < 0.01; *** *p* < 0.001.
